# The Rewards of Institutional Life

**Published:** 1915-01-09

**Authors:** 


### THE REWARDS OF INSTITUTIONAL LIFE.

No profession receives full social sanction for the dignity which its members claim for it until it has achieved a right to have its work rewarded by mention in the annual Honours List. To have done this for the profession of acting was the great achievement of the late Henry Irving, for which he must always claim a place in history whatever opinions be held as to his artistic achievement. In the case of institutional work the issue is somewhat confused by the fact that the various men who receive knighthoods and other honours, while often actively associated in hospital affairs, yet as a rule receive their distinctions for some other reason than their achievement for hospitals. Thus, if we glance round at existing hospital managers, secretaries, and medical officers, we shall find secretaries, superintendents, and medical men all holding titles and distinctions, but hitherto, in a variety of cases, the honour has been acquired for other than hospital service. The profession in its modern sense is so new and its importance so recently recognised that it is, no doubt, natural that men who have achieved distinction in other fields, though often allied fields, should be attracted to it rather than that its own veterans should have been rewarded with titular distinction. Indeed, by the voluntary system, with its army of unpaid workers and deliberate policy of attracting to its service the best brains and energies of the business world, this condition of affairs is fostered. But of late years a pleasing tendency has begun to show itself to recognise the claims to distinction of the chief men who have devoted their lives to hospital work. The test of this is that we now find honours bestowed on the paid, as well as on the voluntary hospital worker.

There is, then, a specially encouraging feature in the New Year's Honours List, which, as we note elsewhere, contains among those singled out for distinction the distinguished chairman of the *Dreadnought* Seamen's Hospital, Mr. Perceval Alleyne Nairne, and the clerk to the Metropolitan Asylums Board, Mr. T. Duncombe Mann. It is a happy augury for the hospital world that the New Year should be marked by the bestowal of knighthoods on representatives of official and voluntary institutional affairs, and no competent person will deny the representative nature and high deserts of the two gentlemen selected for decoration. To their general records, which we give in detail on another page, we need not here refer, but merely note how extensive and constant have the services been in each case.

The material rewards of institutional life, taking the prospects as a whole, are slender, and, as the amount of work exacted by the nature of the posts is extremely large and of immense public value, it should hearten and encourage the rank and file to note the public prestige which in increasing measure the profession is acquiring. Public responsibility and public estimation are good alike for professions and individuals, and therefore we hope that this New Year may also see the beginning of more adequate remuneration within the ranks of the profession itself. Probably of no other occupation could it be truthfully said, as is the case in hospital life, that every class of institutional worker is inadequately paid; whether you look at the nursing staff, the secretariat, or the doctors, the same condition of inadequate remuneration is evident. One class, the doctors, have already begun to make up for lost time, since the war placed a premium upon their services, and we hope and believe that the salaries of the other two classes will shortly show a similar improvement. Probably the nurses will next receive attention, since a move in this direction seems already in evidence, and then the well-deserved turn of the administrative staff will come. The growth of a modern hospital secretary's responsibilities, the great interests which a modern hospital represents—these demand an education, a driving force, a prolonged training, and if the changes ahead of our institutions are to be wisely managed, the men at the head of administrative affairs must be those of first-class ability, and consequently of first-class remuneration. As an earnest of the necessity for securing the best type of men, and of the improved financial prospect which must accompany it, we direct our readers' attention to the New Year's Honours List, feeling sure that, while appreciating the tendency shown therein, they will also realise how much the hospitals owe to the two new knights and others like them, who have borne the burden of institutional work, unrecognised in the past, from which their colleagues as truly as themselves will reap the benefit.

